# Dipeptidyl peptidase 3, a novel protease from *Leishmania braziliensis*

**DOI:** 10.1371/journal.pone.0190618

**Published:** 2018-01-05

**Authors:** Jenny R. Diaz, Cesar A. Ramírez, Paola A. Nocua, Fanny Guzman, José M. Requena, Concepción J. Puerta

**Affiliations:** 1 Laboratorio de Parasitología Molecular, Departamento de Microbiología, Facultad de Ciencias, Pontificia Universidad Javeriana, Bogotá, Colombia; 2 Núcleo de Biotecnología Curauma (NBC), Pontificia Universidad Católica de Valparaíso, Valparaíso, Chile; 3 Centro de Biología Molecular Severo Ochoa (CSIC-UAM), Universidad Autónoma de Madrid, Madrid, Spain; Karl-Franzens-Universitat Graz, AUSTRIA

## Abstract

The increase of leishmaniasis cases worldwide and the emergence of *Leishmania* strains resistant to current treatments make necessary to find new therapeutic targets. Proteases are appealing drug targets because they play pivotal roles in facilitating parasite survival and promoting pathogenesis. Enzymes belonging to the dipeptidyl peptidase 3 (DPP3) group have been described in different organisms such as mammals, insects and yeast, in which these enzymes have been involved in both protein turnover and protection against oxidative damage. The aim of this work was to characterize the structure and function of the *Leishmania braziliensis* DPP3 (LbDPP3) protein as the first step to elucidate its suitability as a potential drug target. Sequence alignment showed 43% of identity between LbDPP3 and its human orthologous (hDPP3) enzyme. Although the modeled protein adopted a globally conserved three-dimensional (3D) structure, structural differences were found in the vicinity of the active site and the substrate binding-cleft. In addition, the *Leishmania* protein was expressed as a soluble recombinant protein and its kinetics parameters were determined using the z-Arginine-Arginine-AMC substrate. The LbDPP3 activity was maximal at pH values between 8.0–8.5. Interestingly, classical enzyme inhibitors such as the tynorphin and its derivative peptide IVYPW were found to actively inhibit the LbDPP3 activity. Moreover, these DPP3 inhibitors showed a detrimental effect upon parasite survival, decreasing the viability of promastigotes by up to 29%. Finally, it was observed that LbDPP3 was equally expressed along the in vitro differentiation from promastigotes to axenic amastigotes. In conclusion, these findings suggest that the *L*. *brazileinsis* DPP3 could be a promising drug target.

## Introduction

Leishmaniasis is a disease caused by several species of the *Leishmania* genus, with a broad range of clinical presentations such as visceral, cutaneous, mucosal and diffuse, according to the infecting species and the immune status of the patient [1–3]. Leishmaniasis is an endemic pathology in several Latin American, Africa, Eastern Europe and East and Central Asia countries [4]. Currently, leishmaniasis is ranked second, after malaria, among parasitic diseases regarding mortality rates [4, 5]. In Latin American countries, *Leishmania braziliensis* is one of the most prevalent causative agent of cutaneous and mucocutaneous leishmaniasis [6]. The most common treatment against leishmaniasis is conventional chemotherapy with pentavalent antimonials such as sodium stibogluconate (Pentostan®) and meglumine antimoniate (Glucantime®). Nonetheless, the use of these drugs has been associated with cardiovascular, renal, hepatic and gastrointestinal failure. In addition, the difficulties of their administration over longer periods and the appearance of resistant parasite strains show the need for developing new therapies [6–9].

During the infection, *Leishmania* spp expresses different virulence factors to invade and maintain the infection in the mammalian host [10–12]. Among them, proteases are relevant for parasite survival as they play a variety of pivotal functions. For instance, the surface glycoprotein Gp63, a zinc-metalloprotease, protects the parasite from the host immune system attack [13, 14], and the oligopeptidase B (OPB), a serine protease that would be involved, once parasites are inside the macrophages, in the degradation of enolase-plasminogen complexes formed on the parasite membrane [15]. The genomes of the different species of *Leishmania* encode for a large number of this class of enzymes: *L*. *major* genome contains 154 genes for proteases that represent 1.8% of its gene content [10]. Similarly, in *L*. *braziliensis*, the protease inventory consists of 44 cysteine proteases, 23 serine proteases and 97 metalloproteases [12]. To date, few of them have been studied [14, 15]. Moreover, it is likely that the inhibition of a sole protease could not be enough to prevent multiplication of *Leishmania* amastigotes [16].

Dipeptidyl peptidase 3 (DPP3) is a metalloproteinase of the M49 family which contains a catalytic center with metal Zn^2+^ as cofactor [17, 18]. This family is characterized by the presence of two different motifs: HELLH and EEXR(K)AE(D), which are involved in the zinc coordination [19, 20]. In species such as human and yeast, it has been described that the main function of DPP3 is to hydrolyze peptides ranging from 3 to 10 amino acids in length from their N-terminal in dipeptides and free amino acids [17, 18, 20]. It has been also shown that DPP3 activity is inhibited by synthetic hermophin-like peptidases in rats and humans [21, 22]. Interestingly, for hDPP3, high resolution crystal structures of the protein in complexes with opioid peptides (Met-and Leu-enkephalin, and endomorphin-2), angiotensin-II and the peptide inhibitor IVYPW have been obtained, showing that differences in the binding modes allow a distinction between real substrates and inhibitory peptides [23]. Those structural data are valuable for a rational design of specific hDPP3 inhibitors.

The DPP3 is mainly located in the cytosol of distinct cell types such as those from human muscle, skin and neuroblastoma; also, it has been found in *Drosophila melanogaster* Schneider S2 cells, and *Saccharomyces cerevisiae*, amongst others [17, 18, 20, 24]. In addition to its role in protein turnover, evidence suggests that DPP3 could be involved in more specific roles such as inhibition of the NF-E2–related factor 2 (NRF2) ubiquitination, which promotes the oxidative damage survival of cancer cells [25].

Proteomic analysis of the *Leishmania donovani* secretome showed the presence of a putative dipeptidyl peptidase 3 in this parasite [26]. However, this protein has not been characterized in any *Leishmania spp*. to date. Thus, the aim of the present work was to characterize the molecular and biochemical features of *L*. *braziliensis* DPP3 as a first approach to elucidating its suitability as a potential drug target.

## Methods

### Ethics statement

The *L*. *braziliensis* MHOM/BR/75/M2904 reference strain was bought in the “Centro Internacional de Entrenamiento e Investigaciones Médicas” (CIDEIM) from Cali, Colombia. All *L*. *braziliensis* handling and *in vitro* procedures were performed in laminar flow booths with biosecurity level 2, according to the resolution N° 008430 from the Health Ministry of the Republic of Colombia. *L*. *braziliensis* genomic DNA and proteins, as well as recombinant DNA and proteins, were handled according to the considerations in the above resolution as well.

### Analysis and 3D modeling of the *L*. *braziliensis* and human DPP3 sequences

The sequences of DPP3 from human (UniProt ID: Q9NY33-1) and *L*. *braziliensis* (LbrM.05.0940) were retrieved from the UniProt (http://www.uniprot.org/uniprot/Q9NY33) database and GeneDB server (http://www.genedb.org/Homepage), respectively. The sequences were submitted to a multiple sequence alignment at the Clustal Omega server (http://www.ebi.ac.uk/Tools/services/web_clustalo/toolform.ebi) and their secondary structures were generated using the ENDscript/ESPript 3.0 server [27]. Additionally, both DPP3 sequences were submitted to the Phyre 2 server [28] to obtain their 3D structure models. In the case of hDPP3, the model constructed was performed by homology using the structure crystal with PDB code 3FVY as template. Afterwards, an energy minimization using the Yasara server (http://www.yasara.org/minimizationserver.htm) was performed. The structures obtained were validated with a Ramachandran plot by the PROCHECK tool, available at Swiss Model web-server (http://swissmodel.expasy.org/). Finally, both structures were compared using the visualization software Pymol, and the root-mean-square deviation (RMSD) was used to compare the structure of both enzymes. Additionally, the distance between the Cα of specific amino acids residues in equivalent positions in both superimposed enzymes (hDPP3 and LbDPP3) was measured using Pymol.

### *Leishmania braziliensis* cultures

Promastigotes of *L*. *braziliensis* MHOM/BR/75/M2904 strain were maintained in Schneider`s insect medium (Sigma Aldrich, Inc., St. Louis, USA) supplemented with 20% heat-inactivated fetal calf serum (FCS) (Eurobio, Inc., Les Ulis, France), and 0.1 μg/ml 6-biopterin (Sigma Aldrich, Inc., St. Louis, USA) as reported elsewhere [29]. Parasite differentiation into axenic amastigotes was initiated by incubation of logarithmic phase promastigotes at 35°C with 5% of CO_2_. Parasites were collected at different times (0, 4, 8, 20 and 48 hours) of differentiation. At indicated time points, a sample of parasites was Giemsa-stained and visualized by optical microscopy, the remaining parasites were centrifuged at 609 g at room temperature for 10 min, and pellets were used for protein extraction.

### Cloning and expression of recombinant LbDPP3

The open reading frame of DPP3 was amplified from *L*. *braziliensis* genomic DNA using the forward primer LbDPP3-F (5’ **GGATCC**ATGTCGCACAATGCGCTTTAC 3’, the target site for the restriction enzyme *Bam*HI is shown in bold) and the reverse primer LbDPP3-R (5’ **AAGCTT**CTACAGCGGGATCTCGC 3’, the target site for the restriction enzyme *Hin*dIII is shown in bold), designed from the annotated sequence of *L*. *braziliensis* LbrM.05.0940 gene (GeneDB). Polymerase Chain Reaction (PCR) was carried out in a final volume of 20 μl containing 1× reaction buffer, 200 μM of dNTP mix, 0.5 μM of each primer, 0.02 units per μl of Phusion Hot Start II Taq polymerase (Thermo Scientific, Inc., Waltham, MA, USA), and 5 ng/μl of genomic DNA and 3% dimethyl sulfoxide (DMSO) (Sigma Aldrich, Inc., St. Louis, USA). An MJ Research PTC-100 DNA thermocycler was used for the PCR reaction with the following amplification profile: 98°C/30 s (initial denaturation), 36 cycles of denaturation at 98°C/10s and annealing/extension for 72°C/1 min, and a final incubation at 72°C for 10 min. All the amplified fragments were resolved on agarose gels, stained with ethidium bromide and visualized under UV exposure. PCR products were cloned, following the manufacturer’s instructions, into the pNZY28 vector using the NZY-blunt PCR cloning kit (NZYtech, Lda. Lisbon, Portugal). After sequencing, the *DPP3* gene was subcloned into the pQE30 expression vector (QIAGEN, Inc., Hilden, Germany), creating the pQLbDPP3 plasmid. To obtain the recombinant LbDPP3 (rLbDPP3), which contains an N-terminal His-tag, thermo-competent *E*. *coli* cells (M15 strain) were transformed with the pQLbDPP3 plasmid. The expression of the protein was induced in a log phase culture (A_600_ = 0.5–0.7) by adding isopropyl β-D-1-thiogalactopyranoside (IPTG) (Invitrogen, Inc., Carlsbad, CA, USA), to a final concentration of 1 mM followed by overnight incubation at room temperature (25°C) with vigorous shaking. The recombinant protein was found to be in inclusion bodies and had to be solubilized using a denaturation buffer, containing 7.5 M urea, 1 M thiourea, 0.1 M NaH_2_PO_4_, and 0.01 M Tris-HCl pH 8–8.5. After cell lysis, cell debris were pelleted by centrifugation at 4696 g for 10 min, and the supernatant was loaded onto a Ni^2+^-NTA-column (QIAGEN, Inc., Hilden, Germany). The column was washed six times with the denaturation buffer (pH 6.5) containing 10 mM imidazole. Subsequently, the protein was eluted with the denaturation buffer (pH 4.5) containing 300 mM imidazole. Alternatively, to obtain the active enzyme, after the protein binding to the column, a refolding procedure was achieved by a decreasing urea gradient in a buffer (pH 6.5) containing 0.1 M NaH_2_PO_4_, 0.01 M Tris-HCl, 10% glycerol, and 10 mM imidazole pH 6.5 buffer. Finally, the protein was eluted in a buffer containing 50 mM NaH_2_PO_4_, 300 mM NaCl, 400 mM Imidazole, 10 mM HEPES, 50 μg/ml heparin, 10% glycerol and 50 mM glycine (pH 4.5) [30]. A little amount of soluble protein was recovered by lysis of bacteria in 0.1 × PBS (pH 8.0–8.5) containing 0.1% Triton X100, and it was used for subsequent assays. After binding to the column bed, the column was washed six times with 1 × PBS 1 (pH 7.0) and 15 mM imidazole. Finally, the protein was eluted with 1 × PBS (pH 7.0), 500 mM imidazole, 10% glycerol, 75 μM ZnCl_2_, and 3 mM DTT. Protein purity of the different preparations was evaluated by SDS-PAGE electrophoresis.

To increase the solubility of the recombinant protein expressed in bacteria, LbDPP3 was expressed as a fusion protein together with the trigger factor (TF), named here as rTF-LbDPP3. For this purpose, the LbDPP3 coding fragment was cloned into the pCold-TF vector (Takara Bio Inc., Kioto, Japón) to generate the plasmid pCLbDPP3. Thermo-competent *E*. *coli* cells (BL21-DE3 strain) were transformed with the pCLbDPP3 plasmid or with an empty plasmid that expresses only the TF protein, used as a negative control for the enzymatic activity assays. An early log phase culture (A_600_ = 0.3) was induced by adding 0.5 mM IPTG and ZnCl_2_ at 75 μM, and further incubated overnight at 15°C with vigorous shaking. Induced *E*. *coli* cells were lysed by vortexing with glass beads (Sigma, product G 3753) in 0.1 × PBS (pH 8–8.5), 0.1% Triton X-100 and 10 μg/ml lysozyme for ten min, the mixture was centrifuged at 4696 g at 4°C for10 min, and the supernatant was used to purify the protein under native conditions by Ni^2+^-NTA-Agarose chromatography. After binding, the column was washed with 1 × PBS (pH 7.4), 0.1% Triton X100, and 20 mM imidazole. The protein was eluted in a solution containing 45 mM Tris-HCl (pH 8.0), 124 mM NaCl, 2.4 mM KCl, 10% glycerol, and 3 mM DTT, followed by the addition of glycerol up to 50%. The rTF-LbDPP3 and the TF control were also obtained by molecular size fractionation with Amicon tubes of 100 kDa for rTF-LbDPP3 and 50 kDa for TF (Merck Millipore Ltd. Co. Cork, IRL). Briefly, after the lysis of *E*. *coli* cells, the supernatant was load into an Amicon tube and centrifugated at 4°C and 1600 g. Then, an equal volume of a solution containing 50 mM Tris-HCl, 1× PBS and 10% glycerol, pH 7.4, was added, and the mixture was spun down under the same conditions. Finally, the non-filtered sample in the case of rTF-LbDPP3 or the flow through for TF, were recovered, glycerol was added up to 50% and stored at -80°C until use. The protein purification steps were monitored by SDS-PAGE electrophoresis and gel staining with Coomassie R-250.

### *Leishmania* protein extraction and Western blotting

For the detection of the endogenous protein expressed by *L*. *braziliensis*, lysates of promastigotes were analyzed by Western blot using a rabbit polyclonal antibody raised against rLbDPP3 produced in the Antibodies Production Service at the Animal Facility of the Centro de Investigación y Desarrollo–CNAG- CSIC (Barcelona, Spain). *L*. *braziliensis* promastigotes were cultured in Schneider’s medium as indicated above and samples were spun down at 609 g for 10 min at 4°C. The pellets were suspended in 0.1× PBS, 0.1% Triton-X100 and subjected to 5 cycles of freezing in liquid nitrogen and thawing at 37°C. The cellular debris were eliminated by centrifugation at 10,000 g for 10 min and the lysate was stored at -80°C. Protein amounts were quantified using the Micro BCA™ protein assay kit (Thermo Scientific, Inc., Waltham, MA, USA) and adjusted by densitometry using a standard curve of known concentration of bovine serum albumin. 13 μg extract were mixed with an equal volume of Laemmli 2× buffer, electrophoresed on 10% SDS-PAGE gels and transferred to nitrocellulose membranes at 200 mA for 1 hour using a buffer containing 25 mM Tris base, 192 mM glycine and 20% methanol. The blots were incubated with blocking solution 1× (Roche, Inc., Mannheim, Germany) in 100 mM maleic acid, 150 mM NaCl, pH 7.5 for 1 hour at room temperature, and then incubated overnight at room temperature with the anti-LbDPP3 rabbit polyclonal antiserum diluted 1: 5,000 in blocking solution. Excess of antibody was washed with 1× PBS, 0.1% Tween-20, 4 times during 10 min each. The membrane was incubated with peroxidase-conjugated anti-rabbit IgG 1:5,000 for 2 hours at room temperature with shaking, and finally washed four times with 1× PBS, 0.1% Tween-20 during 10 min each. Detection was performed by using the SuperSignal West Pico Chemiluminescent Substrate (Thermo Scientific, Inc., Waltham, MA, USA). Densitometric analyses were performed using Quantity One 4.5.0 software.

### Enzymatic activity determinations

Enzymatic activity of the recombinant rLbDPP3 or rTF-LbDPP3 was evaluated with the fluorogenic DPP3 assay kit (BPS Bioscience, Inc., San Diego, CA, USA) according to the manufacturer’s protocol. Briefly, the reaction (100 μl) was performed in black 96-well microplates using 200 ng (1.5 μl) of the rLbDPP3 and 50 ng of the recombinant N-GST-Tag human enzyme (rhDPP3) (1 μl), provided in the kit, as positive control. The Z-Arg-Arg-AMC was used as substrate in a buffer suitable for the enzyme, provided by the kit. The production of 7-amino-4-methylcoumarin (AMC) was measured every 2 min in a fluorometer (Fluostar Optima) at a wavelength of 355 nm for excitation and 460 nm for emission during 1 hour and 30 min. The substrate affinity of rhDPP3 and rLbDPP3 was measured by testing the enzymatic activity in the presence of different Arg-Arg-AMC concentrations, ranging from 5 to 50 μM for rhDPP3 and 20 to 160 μM for rLbDPP3. The concentration of free AMC produced in the enzymatic reaction was assessed using a standard curve with known concentrations of AMC. The Michaelis-Menten constant (Km) and the maximum rate of the reaction (Vmax) were assessed using the models of Monod, plotting the substrate concentration (μM) and velocity of the reaction in nM/min, and the Langmuir equation: substrate concentration (μM) and the ratio between substrate concentration and velocity (S/V, μM(nM/min)^-1^). Briefly, the Vmax was defined as the inverse of the slope of the Langmuir linear regression equation and the Km was calculated multiplying the Vmax by the intersect of the same equation. The turnover number (kcat) was calculated as Vmax/enzyme concentration. The effects of the tynorphin and IVYPW inhibitors (synthesized by the Núcleo de Biotecnología Curauma, Pontificia Universidad Católica de Valparaíso, Chile) on the activity of both human and *L*. *braziliensis* enzymes were also evaluated. For this purpose, the enzymatic activity was performed as described previously, but in the presence or absence of a 3-fold molar excess of each inhibitor dissolved in water. The enzymatic activity assays were repeated twice for the evaluation of kinetic parameters (Km and Vmax), and at least thrice for the inhibition experiments, each measure was done in triplicate.

### Cell viability assays

The viability of the parasites in the presence of inhibitors for DPP3 was assessed by 3-(4,5-dimethylthiazol-2-yl)-2,5-diphenyltetrazolium bromide (MTT) (Sigma Aldrich, Inc., St. Louis, USA) assay. *L*. *braziliensis* promastigotes were cultured in 96-well plates at a density of 4×10^5^ parasites/well in 150 μl of fresh medium containing either tynorphin or IVYPW at 200 μg/ml. After incubation for 24 or 48 hours, 100 μl of RPMI medium without phenol red and 50 μl of MTT at an initial concentration of 4 mg/ml, were added in each well and incubated further at 26°C for 4 hours. The formazan crystals were dissolved in 100% dimethyl sulfoxide (DMSO) and the absorbance measured in a microplate reader at the wavelength of 595 nm. The reduction of the viability in the presence of DPP3 inhibitors was expressed as the percentage of viable cells respect to the parasites grown in the absence of inhibitors but in the presence of medium.

## Results

### Identification of *L*. *braziliensis* DPP3 and comparison with its human orthologous

**Fig 1:**
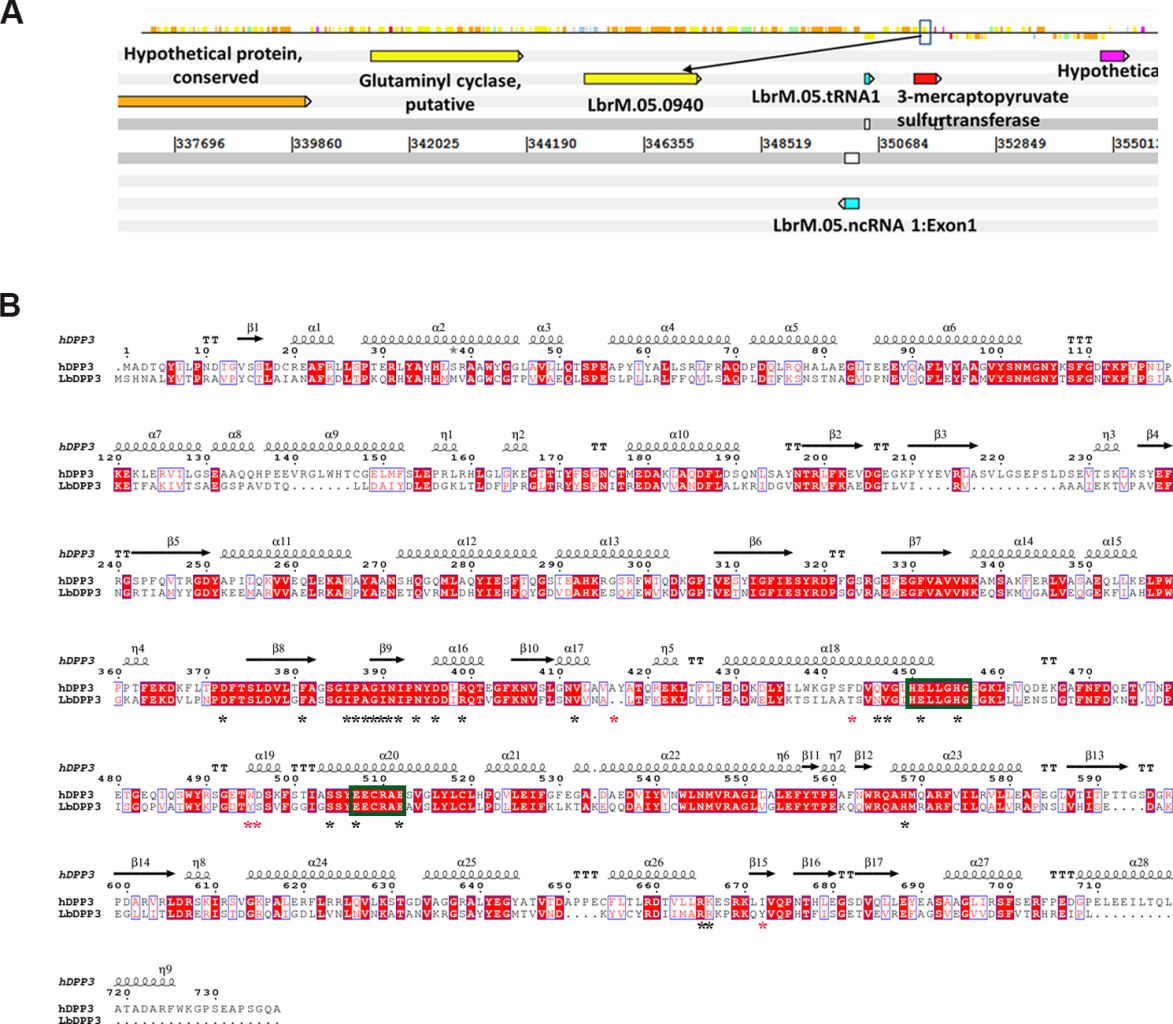
Identification of the gene coding for DPP3 in *L*. *braziliensis* and its sequence conservation. **A**. Localization of DPP3 in the *L*. *braziliensis* genome. Black arrow depicts the DPP3 locus (LbrM.05.0940 gene). Current information on the *L*. *braziliensis* DPP3 Locus at GeneDB database can be accessed through the link **http://www.genedb.org/gene/LbrM.05.0940?actionName=%2FQuery%2FquickSearch&resultsSize=1&taxonNodeName=Root**. **B.** Sequence alignment and secondary structures of the *L*. *braziliensis* (Lb) and human (h) DPP3 enzymes were built using ENDscript 2.0 online tool [27]. Secondary structure elements are represented by squiggles (α-helices), arrows (β-strands) and TT (turns). Red boxes and white characters show strict identity; red characters show similarity in a group: ISc (is a classical computation of a similarity score within each group) > ThIn (threshold for in-Group); blue frame shows similarity across groups: TSc (is the mean of in-Group Score and Cross-Group Score) > ThIn according to ESPript 3.0. Green boxes represent the conserved HELLH and EEXR(K)AE(D) motifs, asterisk show amino acids important for substrate binding to the enzyme, black asterisk amino acids conserved in LbDPP3 and hDPP3 and red asterisks, amino acids different in both enzymes.

**Fig 2:**
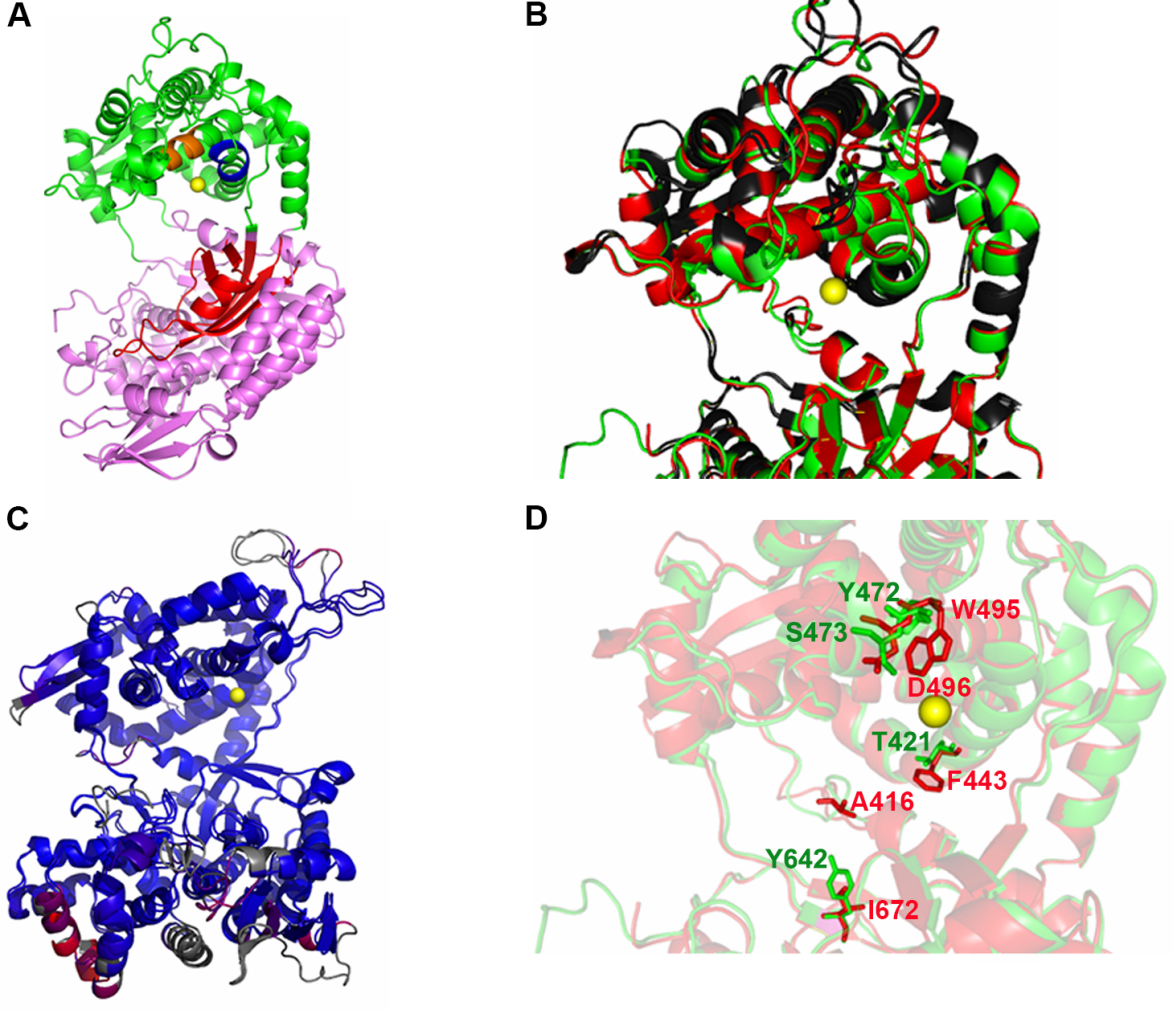
3D modeling of the LbDPP3 and comparison with its human orthologous. **A.** 3D homology model for LbDPP3. The zinc ion is shown in yellow, and the β core in red. The LbDPP3 ^450^HELLGH^455^ and ^507^EECRAE^512^ motifs are shown in orange, and blue, respectively. The upper domain is shown in green and lower in violet. **B.** Superposition of the *L*. *braziliensis* (green) and human (red) DPP3 structures. Amino acids residue differences, showed in Fig 1B, are indicated in black. The image shows a close up of the cleft area and the active site and amino acids important for the substrate binding **C.** RMSD (1.9 Å) by color of LbDPP3 and hDPP3. The blue color shows the good alignment and the red, regions with differences in the conformation of the proteins. Residues not used for alignment are colored in gray. **D**. Important amino acids for the substrate binding with different properties between LbDPP3 (green: T421, Y472, S473 and Y642) and hDPP3 (red: F443, W495, D496, I672). The modeling was performed using Phyre V.2.0 [28] and the visualization and annealing, with the Pymol viewer software.

The *L*. *braziliensis* DPP3 protein was initially identified by mass spectrometry in a study aimed to identify differentially expressed proteins during parasite thermal shock (data not shown). Therefore, to confirm the presence of DPP3 in the *L*. *braziliensis* genome, we searched its complete genome sequence available at GeneDB database. It was found that the protein is coded by the LbrM.05.0940 gene (Fig 1A). In agreement with the *L*. *braziliensis* genome database, Southern blot analysis confirmed that this is a single-copy gene (S1 Fig). Additionally, a multiple sequence alignment was performed using the Clustal Omega program to analyze the sequence conservation between LbDPP3 and its homologous hDPP3, finding a sequence identity of 43% (Fig 1B). The 3D structures of both proteins were modeled using Phyre 2 server [28] (Fig 2). The constructed models showed that both proteins would adopt a similar global structure, with a root-mean-square deviation (RMSD) of 1.9 Angstroms. Whereas LbDPP3 conserves the amino acids of the HELLH and EEXR(K)AE(D) motifs, involved in the formation of the Zn coordination complex, as well as those of the β-secondary structure, known as β-core important for binding of substrate [19, 22] (Fig 2A–2C), some amino acids differences were also observed close to the enzyme active site and the cleft where the substrate binds (Fig 2D). In addition, some amino acids that have been characterized as critical for the correct binding of substrate to hDPP3 [22], also differed in their chemical properties (Fig 2E and Table 1). Moreover, after the structural alignment of the 3D structures, distance differences between the Cα of these amino acids, which have equivalent positions in both proteins, were observed. For instance, the W495/hDPP3 and Y472/LbDPP3 amino acids as well as D496/hDPP3 and S473/LbDPP3 (Table 1).

**Table 1:** Relevant amino acids for substrate binding that are different in LbDPP3.

	Protein	Amino acids	Ref.	Type of amino acids	Cα distance between aa pair*
**1**	hDPP3	F443	[22]	Aromatic, nonpolar	0.5 Å
	LbDPP3	T421		polar, uncharged	
**2**	hDPP3	W495	[22]	Aromatic	1.1 Å
	LbDPP3	Y472		Aromatic, polar	
**3**	hDPP3	D496	[22]	Acidic	1.1 Å
	LbDPP3	S473		Polar	
**4**	hDPP3	I672	[22]	non-polar, aliphatic	0.2 Å
	LbDPP3	Y642		Aromatic, polar	
**5**	hDPP3	A416	[22]	nonpolar, aliphatic	___
	LbDPP3	-		-	

* The greater the value, the greater the difference

### Identification of the endogenous *L*. *braziliensis* DPP3 enzyme

**Fig 3:**
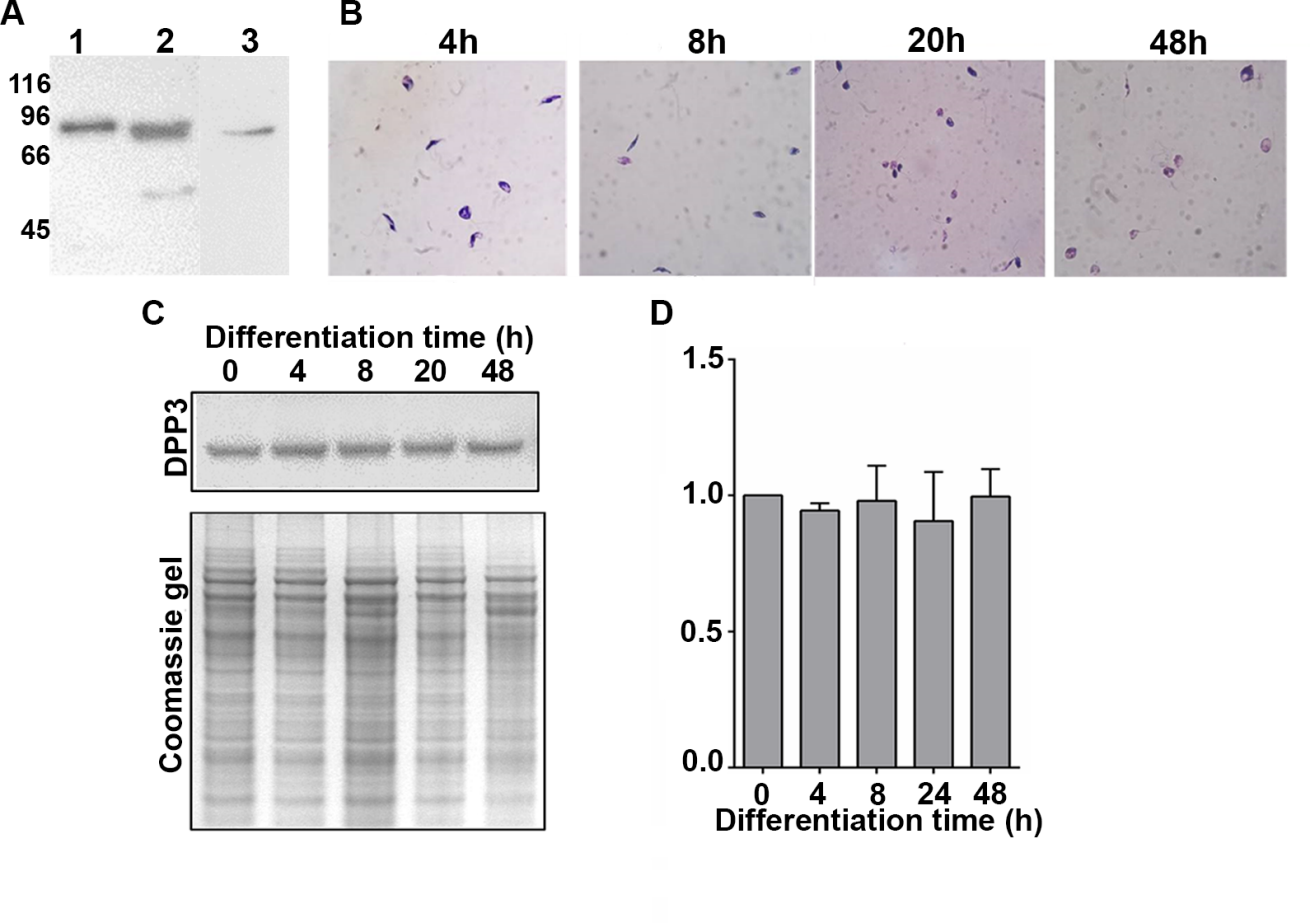
Endogenous expression of DPP3 in *L*. *braziliensis*. **A.** 300 ng of rLbDPP3 (lane 1) or 13 **μ**g of protein extract from *L*. *braziliensis* promastigotes (lanes 2 and 3) were separated by SDS-PAGE gel at 12% and blotted onto a nitrocellulose membrane. The blot was incubated with a polyclonal antibody raised in a rabbit against the rLbDPP3 (lanes 1 and 2) and with the same antibody after its purification by affinity to the rTF-LbDPP3. **B.** Giemsa staining of parasites grown at 0, 4, 8, 20 and 48 hours in promastigote-to-amastigote axenic differentiation conditions. **C.** Western blot of *L*. *braziliensis* lysates obtained at different time points during the promastigote-to-amastigote differentiation process. As control of protein load, a Coomassie blue stained SDS-PAGE gel with the same samples is shown. **D.** DPP3 levels, in arbitrary units, normalized to the total protein observed in Coomassie blue stained gel, as determined by densitometric analyses.

To characterize the endogenous DPP3 of *L*. *braziliensis*, a Western blot was performed with a rabbit antiserum raised against the rLbDPP3, which was expressed in *E*. *coli* and purified by affinity chromatography using Ni-NTA columns. The antiserum recognized around 77-kDa, the recombinant (Fig 3A, Lane 1) as well as the native protein expressed in *L*. *braziliensis* promastigotes (Fig 3A, Lane 2). Also, in *L*. *braziliensis* promastigotes it was detected a second signal of ≈52 kDa which disappeared when the Western blot assay was performed using the polyclonal anti-rLbDPP3 antibody previously purified by affinity against the rTF-LbDPP3 protein (Fig 3A, Lane 3).

To evaluate the expression of the *L*. *braziliensis* endogenous protein during the *in vitro* promastigote to amastigote differentiation process, *L*. *braziliensis* promastigotes were cultured at 35°C and 5% of CO_2_ for up to 48 hours, time in which most of the parasites adopted the amastigote shape (Fig 3B). The levels of DPP3 expression were evaluated by Western blot at 0, 4, 8, 20 and 48 hours after starting the differentiation process. The recollection times were chosen because of the shapes that the parasites develop at each time point: at 4 hours the shape of the parasites was promastigotes-like, at 8 hours, some parasites had rounded shape, at 20 hours, most parasites were rounded and some of them lost the flagella and finally at 48 hours, most of the parasites were amastigote-like, but some of them still presented flagella. The data were normalized versus the amount of total protein on each lane, estimated by densitometry of the SDS-PAGE gel stained with Coomassie. The results showed that during the process of metacyclogenesis, the protein was expressed at the same levels (Fig 3C and 3D).

### Cloning and expression of the rLbDPP3

After cloning into the pQE30 expression vector, the recombinant protein (rLbDPP3) was expressed in *E*. *coli* M15 using IPTG and ZnCl_2_ to induce its expression. As shown in S2 Fig, compared with the non-induced culture (Lane NI), a strong band of approximately 77 kDa was observed in the induced culture (Lane I) and in the soluble and insoluble fractions of the sonicated induced cultures (Lanes S1-S3 and Insoluble). Furthermore, the protein was purified by Ni/NTA affinity chromatography under denaturing conditions (S2 Fig) and used to obtain the polyclonal anti-rLbDPP3 serum.

**Fig 4:**
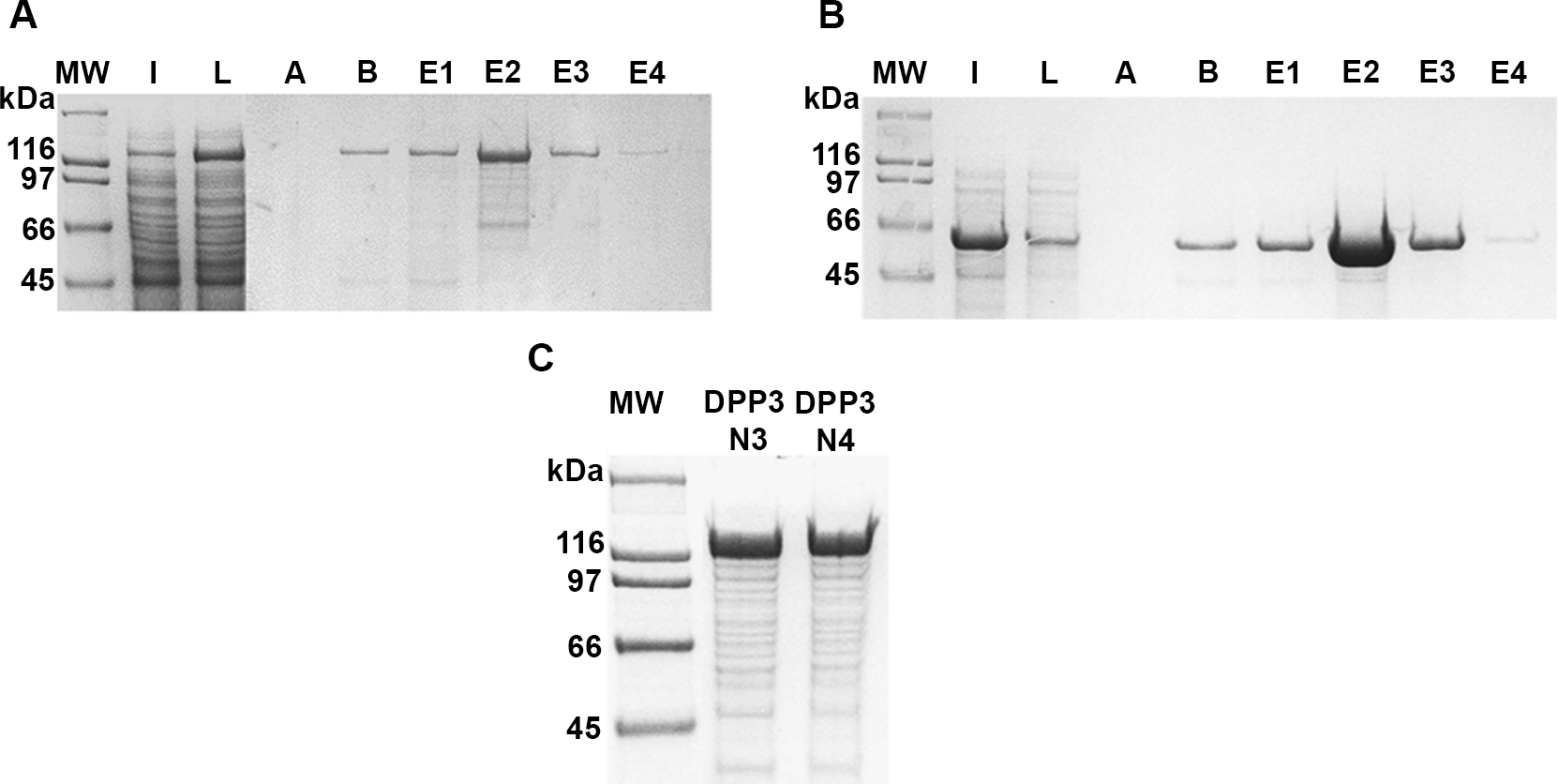
Expression of the rTF-LbDPP3 protein in bacteria transformed with the pCLbDPP3 plasmid. **A.** Induction of the expression of rTF-LbDPP3 in *E*. *coli* BL21, followed by lysis with 0.1× PBS and 0.1% Triton X100 buffer and isolation of the enzyme by Ni-NTA affinity chromatography. **B.** Expression of TF protein in *E*. *coli* BL21, under the same condition as rTF-LbDPP3. **C.** After lysis of *E*. *coli* cells, two batches of the native rLbDPP3 were obtained using Amicon tubes of 100 kDa without purification for affinity chromatography. MW: Molecular Weight; I: induction of the protein expression; L: Soluble protein fraction after *E*. *coli* lysis by sonication; A and B, successive washes after protein binding; E1-E4: successive elutions; N3-N4, protein extract remaining after filtering with Amicon tubes.

In order to study its enzymatic activity, the rLbDPP3 was obtained in a soluble form by two methods: first, by the Ni-NTA-column refolding process of the insoluble bacteria lysate material, and second, by the Ni-NTA purification of the small amount of protein present in the soluble fraction. Unfortunately, the protein obtained by these two methodologies showed a very low or nonexistent enzymatic activity (S3 Fig). After modeling the 3D structure of the rLbDPP3 protein, it was shown that the low activity could be derived for a structural interference caused by the N-terminal His-tag within the LbDPP3 catalytic site (S4 Fig). Therefore, we cloned the LbDPP3 gene into the pCold-TF expression plasmid, as the modeling of the TF-LbDDP3 did not show any structural interference between the TF factor and the catalytic site of the enzyme (S5 Fig). Moreover, this fusion protein, having the expected size of 129 kDa, was recovered from the soluble fraction (Fig 4). Also, to be used as control, the TF protein alone, derived from the pCold-TF plasmid, was expressed and purified in the same conditions as the rTF-LbDPP3.

### Analysis of the enzymatic activity of *L*. *braziliensis* recombinant DPP3

**Fig 5:**
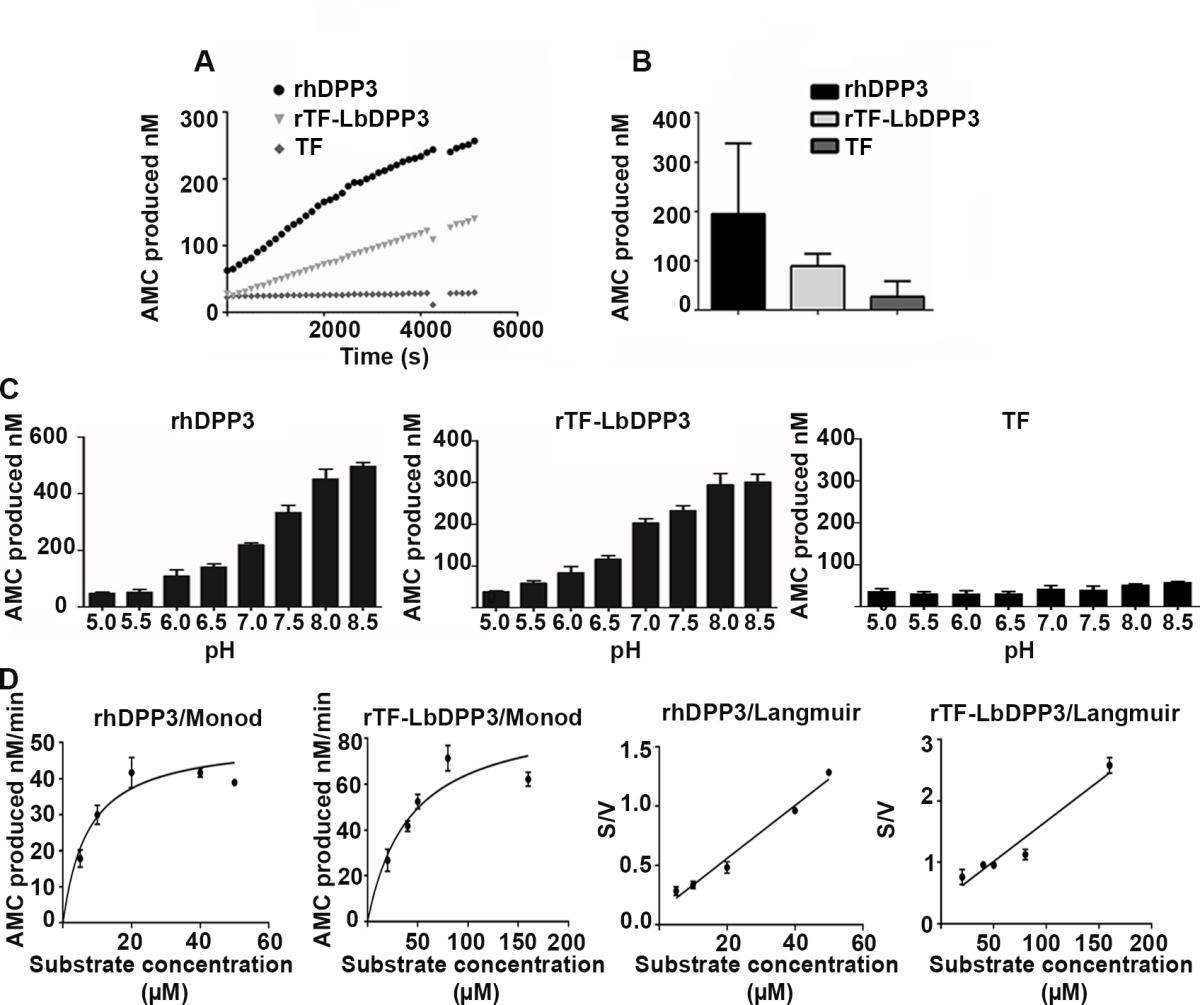
Enzymatic activity of rTF-LbDPP3. **A.** AMC (nM) produced by rTF-LbDPP3 purified by amicon tubes, rhDPP3 (positive control) and TF (negative control) along 80 min of reaction. **B.** AMC (nM) produced after 45 min of reaction. Each experiment was done in triplicate. **C**. Enzymatic activity was evaluated at different pH values, from 5.0 to 8.5, for hDPP3, rTF-LbDPP3, and TF. Each experiment was done in triplicate after 45 min of enzymatic reaction. **D**. The Vmax and Km were evaluated using different concentrations of substrate ranging from 1.25 to 50 μM for hDPP3 and from 1.25 to 160 μM for rTF-LbDPP3. Left panels show the Monod model and right panels the Langmuir model for each enzyme. The enzymatic activity was measured as AMC produced per minute of reaction. The values of the negative control (TF protein), measured in the same conditions, were subtracted from the values obtained for the rTF-LbDPP3 enzyme.

After purification of the fusion protein rTF-LbDPP3, the enzymatic activity of this enzyme was determined and compared with the activity of the recombinant human orthologous using the same experimental conditions. The enzymatic activity of rTF-LbDPP3 was analyzed in the fractions obtained by affinity chromatography (Fig 4A) or purify by molecular size fractionation (Fig 4C), as well as the negative control TF purified (Fig 4B). Interestingly, the rTF-LbDPP3 showed a remarkable activity when assayed with the Z-Arg-Arg-AMC substrate, which is highly specific for the human DPP3 protein (Fig 5). However, the activity of rTF-LbDPP3 obtained by Ni^+^ affinity chromatography was found to be clearly lower than that of the protein purified by molecular size (S6 Fig).

The optimum pH for enzymatic activity of *L*. *braziliensis* and human enzymes was determined by measuring the production of AMC for each enzyme in the pH range between 5–8.5. It was observed that hDPP3 and LbDPP3 have optimal activity at basic pH values: hDPP3 at 8.5 and rTF-LbDPP3 at 8.0–8.5 (Fig 5C). Additionally, it was found that rTF-LbDPP3 presented less affinity for the substrate Z-Arg-Arg-AMC (Km of 26.5 μM) than hDPP3 (Km of 4.89 μM) (Fig 5). On the contrary, theVmax was higher for LbDPP3 (75.55 nM/min) than that for hDPP3 (44.84 nM/min), which is concordance with the higher concentration of rTF-LbDPP3in the enzymatic reaction. Finally, we found that hDPP3 is a more efficient enzyme with a Kcat of 2.9 s^-1^ for rTF-LbDPP3 and 9.79 s^-1^ for hDPP3.

**Fig 6:**
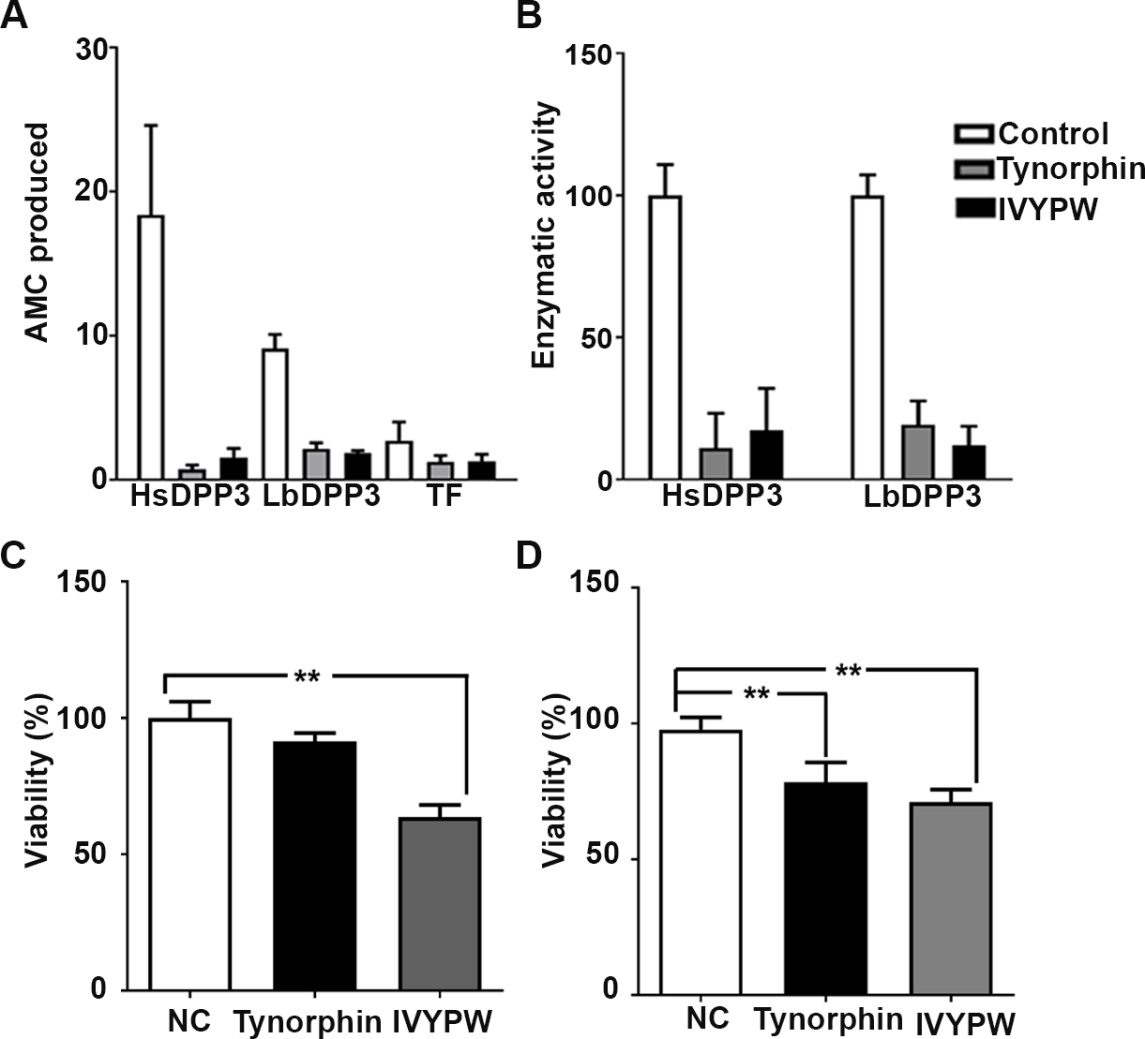
Effect of human DPP3 inhibitors on both the *L*. *braziliensis* DPP3 enzymatic activity and parasite viability. **A.** AMC (nM) produced by either the rhDPP3 (positive control), the rTF-LbDPP3purified by amicon tubes and the TF (negative control), after 45 min of reaction in the presence or absence of either tynorphin or IVYPW inhibitors. **B.** Enzymatic activity inhibition expressed as a percentage of the activity shown in the absence of inhibitors, determined after 45 min of reaction. For determining the rTF-LbDPP3 activity, the values of AMC production in control assay (rTF protein) were subtracted from the values observed in the rTF-LbDPP3 assays. **C**. Inhibitor effect of 200 μg/ml tynorphin or IVYPW on parasite viability, measured by MTT assay at 24 hours. **D**. Inhibitor effect of 200 μg/ml tynorphin or IVYPW on parasite viability, measured by MTT assay at 48 hours. Results were normalized based upon the negative control (NC), corresponding to parasites in medium without treatment but in presence of the vehicle (water). Significant differences were shown between NC and tynorphin or IVYPW 200 μg/ml for 48 hours and between NC and IVYPW 200 μg/ml for 24 hours. ** *p < 0*.*01* analyzed by Mann-Whitney test.

In addition, we evaluated the effect in the LbDPP3 activity of well-known inhibitors of the hDPP3 enzyme. Interestingly, it was found that either tynorphin or its derivative IVYPW produced a dramatic decrease in the enzymatic activity of both rhDPP3 and rTF-LbDPP3 (Fig 6).

### Anti-leishmanial activity of DPP3 inhibitors

To elucidate if the LbDPP3 enzymatic activity was relevant for parasite survival in axenic culture, the effects on promastigote viability of tynorphin and IVYPW inhibitors were evaluated. It was observed that the tynorphin (*p = 0*.*0012)* and IVYPW (*p = 0*.*0043)* treatments at 200 μg/ml during a period of 48 hours had a detrimental effect on the promastigotes, reducing their viability by 21.61% and 28.9%, respectively (Fig 6D). However, the tynorphin treatment at 200 μg/ml for 24 hours did not show a significant (*p = 0*.*30)* effect whereas the IVYPW at 200 μg/ml treatment for 24 hours reduced the viability by 29% (*p = 0*.*0043)* (Fig 6C).

## Discussion

Previous studies have reported the relevance of DPP3 in different organisms, from yeasts to humans, in which the protein has been involved in protein turnover, degrading peptides (3–10 amino acids in length) into dipeptides and free amino acids that are used for energy production [17]. Additionally, DPP3 has been implicated in the protection of cancer cells from oxidative damage by a mechanism that inhibits the NRF2 ubiquitination [24]. However, the role of this enzyme has not been studied in any *Leishmania* species to date. Nevertheless, it is important to mention that this protein was found in the secretome of *L*. *donovani*, one of the species that causes visceral leishmaniasis [26]. Due to the crucial functions of DPP3 in other organisms, the availability of specific inhibitors [21, 22, 31, 32], and its confirmed presence in other relevant *Leishmania* species (*L*. *donovani*); the aim of this work was to characterize the *L*. *braziliensis* DPP3 by bioinformatics tools, and biochemical and enzymatic assays, to evaluate its potential as a therapeutic target.

The sequence analysis of DPP3 in the *L*. *braziliensis* genome allowed the identification of a single gene copy that expresses a protein of 679 amino acids in length. Also, we determined the presence of the endogenous *L*. *braziliensis* DPP3 and evaluated its expression along the *L*. *braziliensis in vitro* differentiation process from promastigote to axenic amastigotes. The results showed that the parasite expresses a DPP3 protein of ≈77-kDa with a constitutive expression along the promastigote-to-amastigote differentiation process.

A search for trypanosomatid homologous proteins in the Tritryp database (http://tritrypdb.org/tritrypdb/app/record/gene/LbrM.05.0940#orthomcl_link) revealed that *DPP3* gene is present in the genome of *Leishmania*, *Endotrypanum*, *Crithidia*, *Leptomonas* and *Blechomonas* but not in any of the species of the genus *Trypanosoma* analyzed to date (S1 Table). Interestingly, regarding *Leishmania* species, this gene is present in both, human’s infectious species and species infecting other mammals, having their translated amino acids sequences an identity of 88–89% with that of the *L*. *braziliensis* enzyme. Similarly, in other trypanosomatids which are also digenetic parasites, including the *Endotrypanum* [33] and *Blechomonas* genus [34], the *L*. *braziliensis* DPP3 sequence has 80% and 65% of identity, respectively (S7 Fig). In addition, the DPP3 predicted sequences of the *Crithidia* and *Leptomonas* genera, which only infect invertebrate hosts [34], also share with LbDPP3 a sequence identity of 80%. These data suggest that DPP3 would be required in both the invertebrate and mammalian parasite stages. Remarkably, *Leishmania tarentolae*, which is not pathogenic to humans [35], encodes a truncated DPP3 protein of 156 amino acids (equivalent positions to LbDPP3 524–679), containing only six of the 35 important sites for substrate binding and zinc coordination.

The expression of the *L*. *braziliensis* DPP3 as a recombinant protein in bacteria did not result in an easy task. Indeed, in our case, most of the rLbDPP3 protein produced by *E*. *coli* formed insoluble polypeptide aggregates as inclusion bodies. For this reason, we assayed a method previously reported [30] to solubilize the recombinant *L*. *braziliensis* RPA-1 protein by an in-column refolding method. However, the protein obtained after the process of refolding using a gradient of urea showed very low enzymatic activity. Three-dimensional modeling of the recombinant LbDPP3 pointed out a possible structural interference of the N-terminal His-Tag within the catalytic site. In fact, negligible activity was also obtained when the rLbDPP3 was directly purified from the soluble fraction. Thus, we looked for an alternative strategy for the expression of the recombinant protein. After modeling analysis, it was predicted that cloning of the gene into the plasmid pCold-TF would give an active enzyme, because it expresses a sequence for a chaperone protein which carries at its N–end the His-Tag, then overcoming the structural hindrance of the His-Tag on the active site of the enzyme. Indeed, following this strategy, it was possible to obtain a soluble and functional enzyme.

The *L*. *braziliensis* DPP3 deduced amino acids sequence contained the conserved HELLGH and EEXR(K)AE(D) motifs critical for the coordination of the zinc ion, that constitute the catalytic center of the enzyme (Fig 2A) [17, 19, 23]. In addition, the 3D structural model showed the presence of the characteristic upper and lower domains, separated by a cleft highly conserved, where the substrates bind (Fig 2A). However, there were found notable differences in the sequence of both proteins, more specifically in the type of amino acids residues close to the cleft, and in amino acids reported previously as key for substrate binding to hDPP3 (Fig 2B and 2D) [19, 22, 23]. Moreover, after superposition of both enzymes 3D models, distance variations between the carbons alpha of relevant amino acids for the substrate binding were observed on the amino acids pairs W495/hDPP3 and Y472/LbDPP3, D496/hDPP3 and S473/LbDPP3, F443/hDPP3 and T421LbDPP3, and I672/hDPP3 and Y642/LbDPP3 (Table 1).

In agreement with the 3D-modeling, which showed that LbDPP3 has the crucial features required for having enzymatic activity, the rTF-LbDPP3 enzymatic activity was determined by its ability to hydrolyze the Z-Arg-Arg-AMC substrate. Nonetheless, we demonstrated a differential activity between the human and *L*. *braziliensis* DPP3 enzymes, which could be attributed to the amino acids differences indicated above, especially those related to the substrate binding cleft. Certainly, a differential affinity and efficiency between the human and *L*. *braziliensis* enzymes for the Arginine-Arginine-AMC substrate were found, where rTF-LbDPP3 presented a higher Michaelis Menten constant (Km) and a lower Kcat.

As the enzymatic activity depends upon several factors such as the optimum pH [36], we evaluated the pH-dependence of the LbDPP3 and hDPP3 enzymes. We found, in our experimental conditions, that both have their highest activity at basic pH, approximately 8.5 for human DPP3, in agreement with a previous report showing an optimal pH between 8.5–9.0 [17], and 8.0–8.5 for the *L*. *braziliensis* enzyme. According to this result, it is plausible that the LbDPP3 enzyme would be acting inside the parasite rather than extracellularly. Thus, the macrophage parasitophorous vacuole exhibits an acid pH [37], and the insect vector gut, even though undergoes an alkalization process after blood ingestion, remains acid because of a process elicited by *Leishmania* to favor its development [38].

To assess whether rTF-LbDPP3 is affected by the inhibitors reported for DPP3 in other species, tynorphin and its derivative peptide IVYPW were evaluated. Both showed similar inhibitory activity against rTF-LbDPP3 and hDPP3. Accordingly, a detrimental effect of these inhibitors on the survival of promastigotes was observed. Our results, together with the report of the DPP3 overexpression in pentavalent antimony resistant strains [39], suggest a significant role of this enzyme in *Leishmania* parasites survival in the vector or host environments; probably through the peptide degradation, which frees amino acids that will be used, in turn, to energy production in stressful situations.

In conclusion, the *L*. *braziliensis* DPP3 enzyme has been characterized and the recombinant protein could be used to search specific inhibitors that eventually would represent drugs for the treatment of leishmaniasis.

## Supporting information

S1 Fig Southern blot of genomic DNA of *L*. *braziliensis* promastigotes. After digestions with the different restriction enzymes, the expected bands were obtained. *Ava*I: two bands one of 1431bp and other of 711 bp; *Xmn*I: two bands one at 4861 bp and another of 1841 bp; *Xba*I and *Eco*RI: a band bigger than 9057 bp.
(TIF)

S2 Fig Expression and purification of the protein rLbDPP3. **A.** Induction of the expression of rLbDPP3 in *E*. *coli* M15 with 1mM of IPTG. The lysis of *E*. *coli* was performed in denaturing conditions **B.** Obtaining process of rLbDPP3 in Ni/NTA column. The elution 1 (E1) was used to generate the antibodies against the *L*. *braziliensis* DPP3. MW: molecular weight, NI: non-induced culture, I: Induced culture, NR: not retained, E1-E6: Elutions, R: protein retained in resin, S1-S3: soluble protein obtained in sonication 1–3.
(TIF)

S3 Fig Enzymatic activity of rLbDPP3. The enzymatic activity of rLbDPP3 cloned into pQE30, was lower than the activity of the human enzyme, hDPP3, used as positive control.
(TIF)

S4 Fig Structure of rLbDPP3 with His-tag. In magenta are shown the amino acids MRGSHHHHHH belonging to the His-tag generated by the plasmid pQE30. The active site of the enzyme includes the zinc ion (yellow) and the conserved motifs HELLGH and EECRAE that coordinate the ion (red and orange, respectively). The images were visualized with the Pymol software.
(TIF)

S5 Fig Modeling of the enzyme DPP3 generated with the plasmid pCLbDPP3. **A.** Protein modeling of the enzyme cloned into the plasmid pCold-TF which consists of a fusion protein composed of a chaperone protein (brown) of 48 kDa in whose N-terminal there is a 6xHis-tag (magenta). The site for the cleavage of the fusion protein, with the enzyme HRV3c, is shown in cyan, the HELLGH and EECRAE motifs are in red and orange, respectively, and the zinc ion is in yellow. **B.** Protein modeling after the cleavage with the enzyme HRV3c. The amino acids remaining that do not belong to the DPP3 structure, are in magenta.
(TIF)

S6 Fig Enzymatic activity of rTF-LbDPP3. The enzyme rTF-LbDPP3 was expressed using the plasmid pCold-TF and purified by two distinct methodologies: Ni^2+^ chromatography or by molecular size using Amicon tubes. After purification, glycerol up to 50% was added and the enzymatic activity of each one was measured as the production of AMC from the Z-Arg-Arg-AMC substrate.
(TIF)

S7 Fig Multiple sequence alignment of the human (h) DPP3 enzyme, *L*. *braziliensis* (Lb) and *L*. *tarentolae*. The boxes represent the conserved HELLGH and EECRAE motifs. Colors are used to represent the important amino acids for substrate binding as described previously (23).
(TIF)

S1 Table Presence of the gene encoding DPP3 enzyme in other trypanosomatids. (PDF)
