# Supplementary figures and images for: Dipeptidyl peptidase 3, a novel protease from Leishmania braziliensis
Source: PLoS One. 2018 Jan 5;13(1):e0190618. doi: 10.1371/journal.pone.0190618 (PMC5755878; doi:10.1371/journal.pone.0190618)

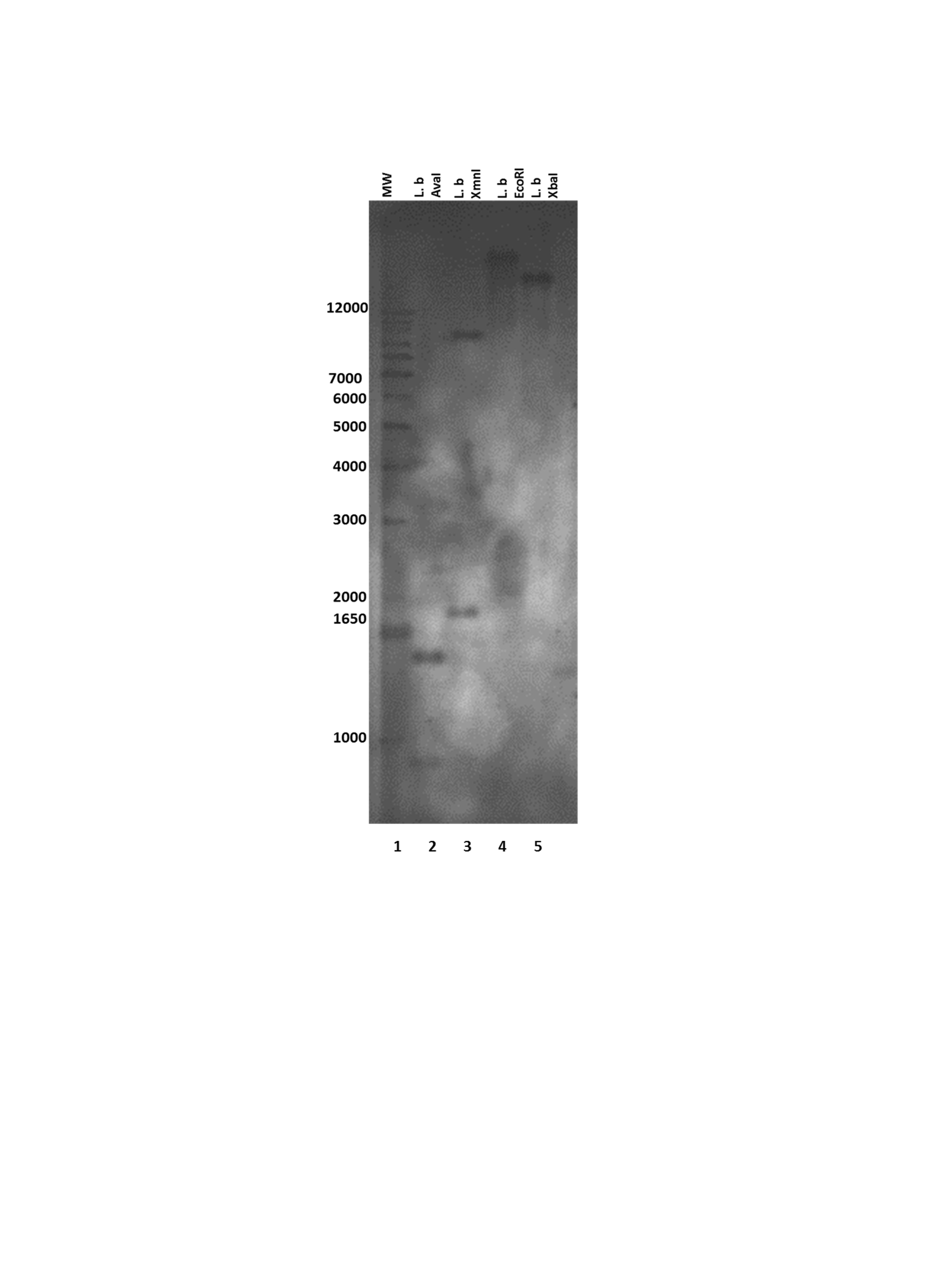

Supplement: S1 Fig — After digestions with the different restriction enzymes, the expected bands were obtained. AvaI: two bands one of 1431bp and other of 711 bp; XmnI: two bands one at 4861 bp and another of 1841 bp; XbaI and EcoRI: a band bigger than 9057 bp. (TIF) [file pone.0190618.s001.TIF]

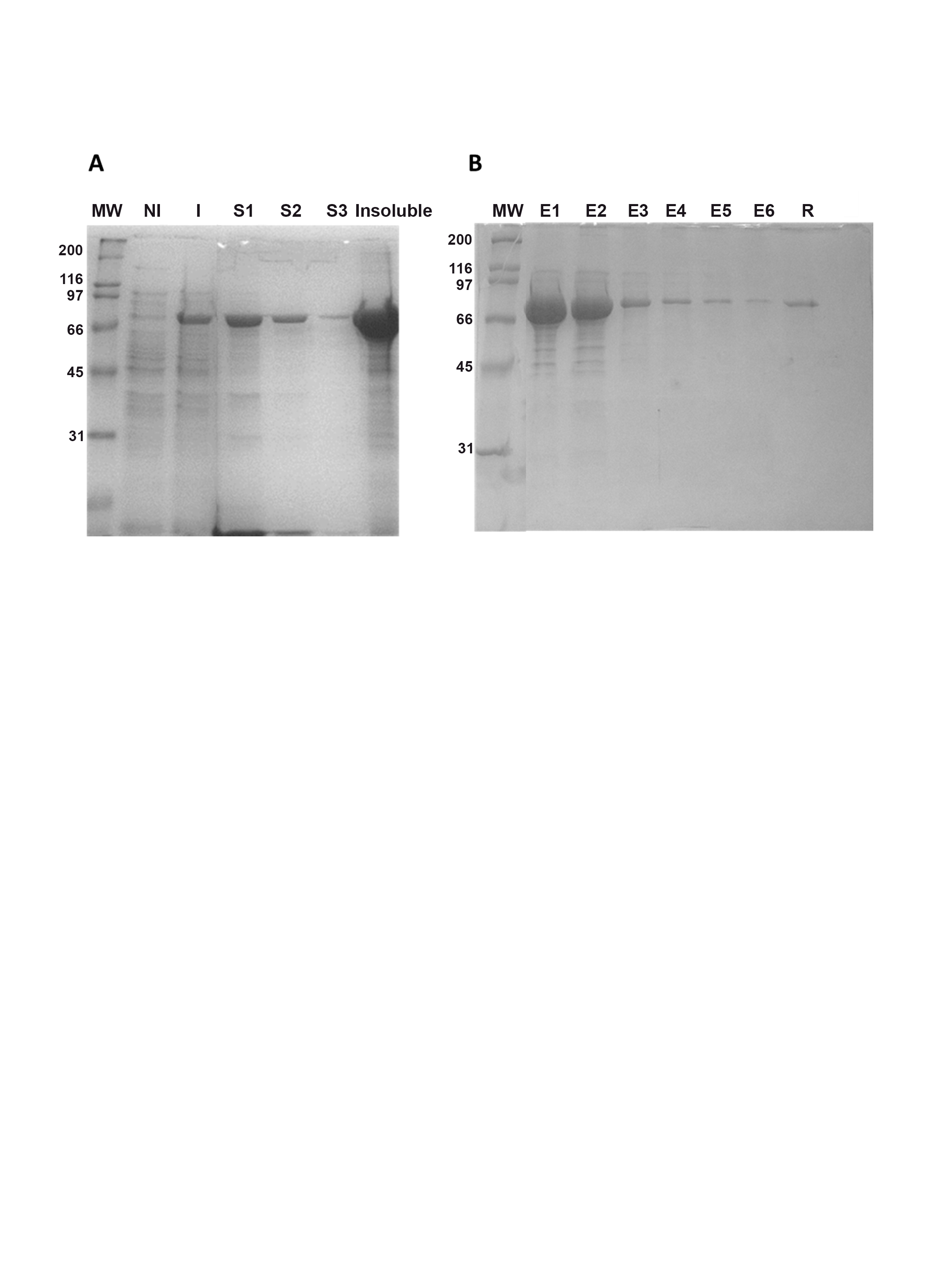

Supplement: S2 Fig — A. Induction of the expression of rLbDPP3 in E. coli M15 with 1mM of IPTG. The lysis of E. coli was performed in denaturing conditions B. Obtaining process of rLbDPP3 in Ni/NTA column. The elution 1 (E1) was used to generate the antibodies against the L. braziliensis DPP3. MW: molecular weight, NI: non-induced culture, I: Induced culture, NR: not retained, E1-E6: Elutions, R: protein retained in resin, S1-S3: soluble protein obtained in sonication 1–3. (TIF) [file pone.0190618.s002.tif]

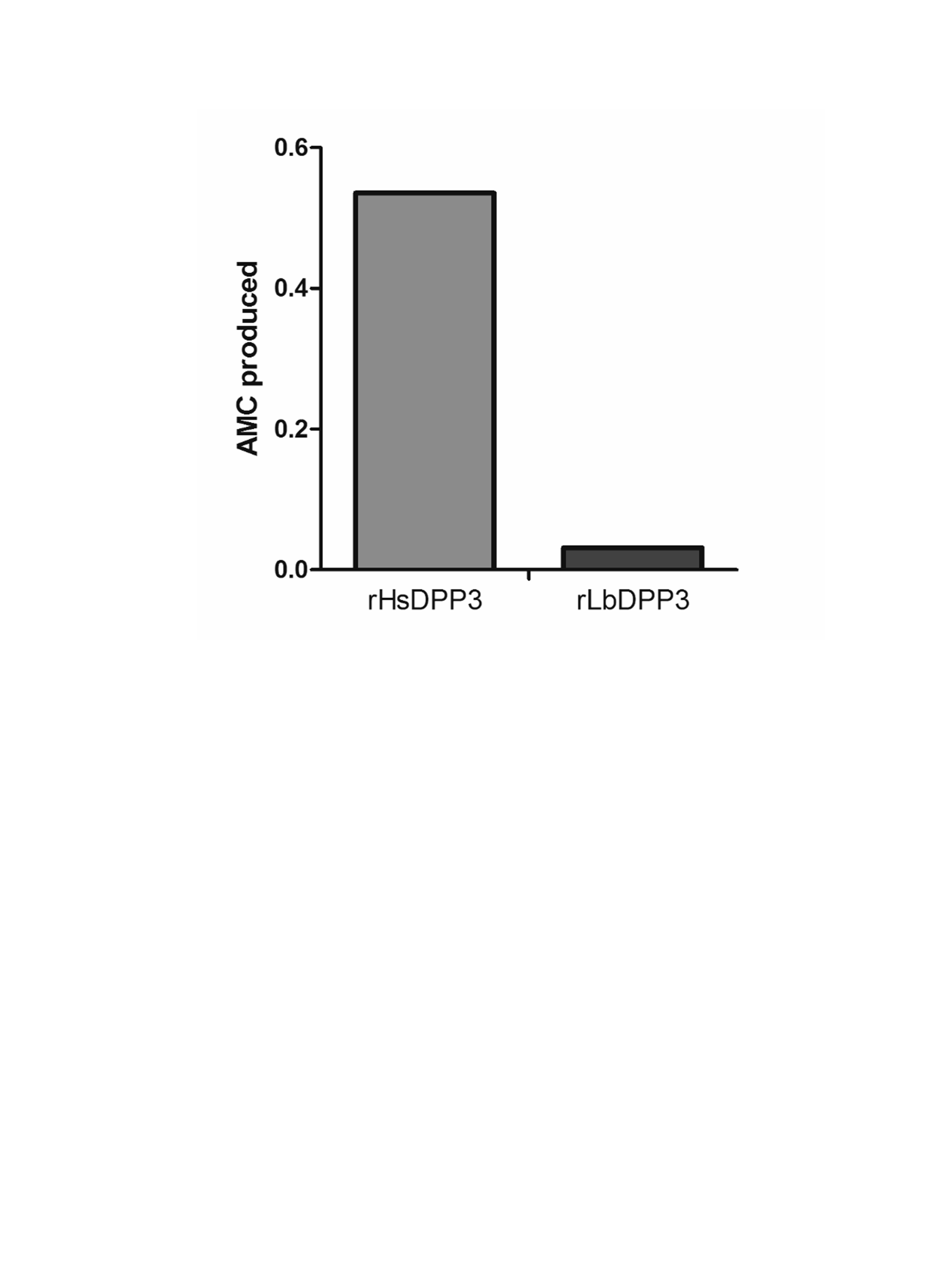

Supplement: S3 Fig — The enzymatic activity of rLbDPP3 cloned into pQE30, was lower than the activity of the human enzyme, hDPP3, used as positive control. (TIF) [file pone.0190618.s003.TIF]

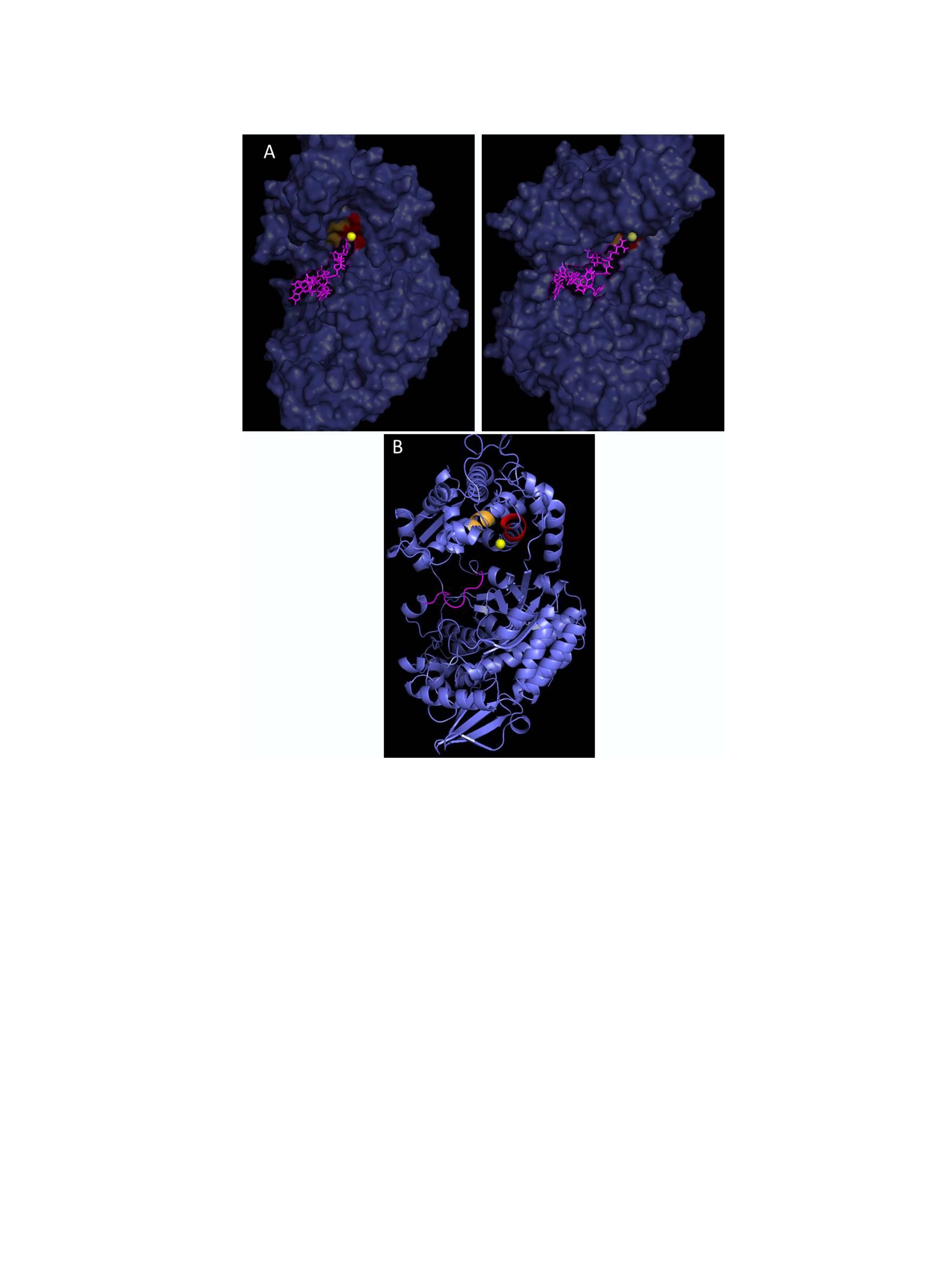

Supplement: S4 Fig — In magenta are shown the amino acids MRGSHHHHHH belonging to the His-tag generated by the plasmid pQE30. The active site of the enzyme includes the zinc ion (yellow) and the conserved motifs HELLGH and EECRAE that coordinate the ion (red and orange, respectively). The images were visualized with the Pymol software. (TIF) [file pone.0190618.s004.TIF]

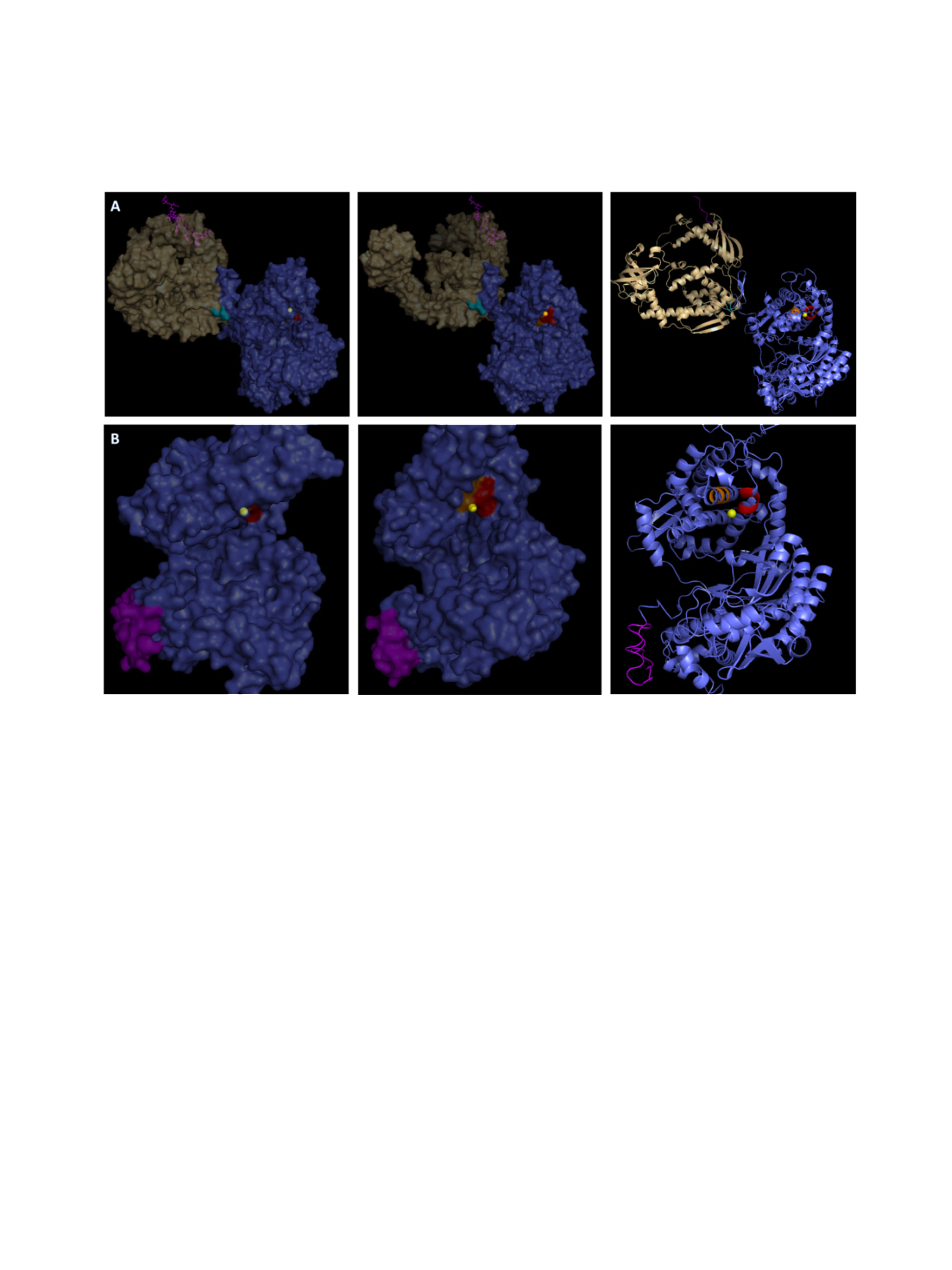

Supplement: S5 Fig — A. Protein modeling of the enzyme cloned into the plasmid pCold-TF which consists of a fusion protein composed of a chaperone protein (brown) of 48 kDa in whose N-terminal there is a 6xHis-tag (magenta). The site for the cleavage of the fusion protein, with the enzyme HRV3c, is shown in cyan, the HELLGH and EECRAE motifs are in red and orange, respectively, and the zinc ion is in yellow. B. Protein modeling after the cleavage with the enzyme HRV3c. The amino acids remaining that do not belong to the DPP3 structure, are in magenta. (TIF) [file pone.0190618.s005.TIF]

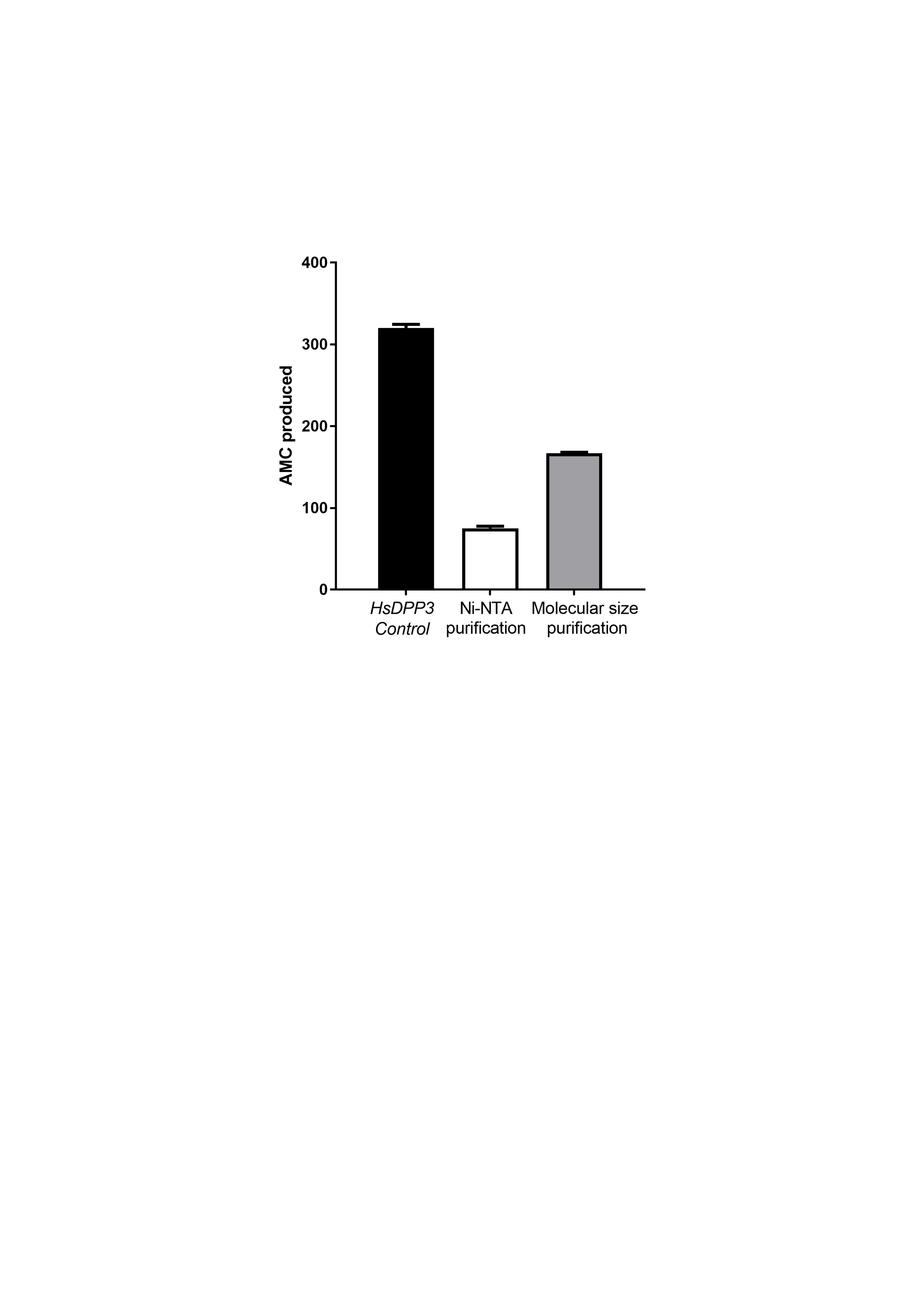

Supplement: S6 Fig — The enzyme rTF-LbDPP3 was expressed using the plasmid pCold-TF and purified by two distinct methodologies: Ni2+ chromatography or by molecular size using Amicon tubes. After purification, glycerol up to 50% was added and the enzymatic activity of each one was measured as the production of AMC from the Z-Arg-Arg-AMC substrate. (TIF) [file pone.0190618.s006.TIF]

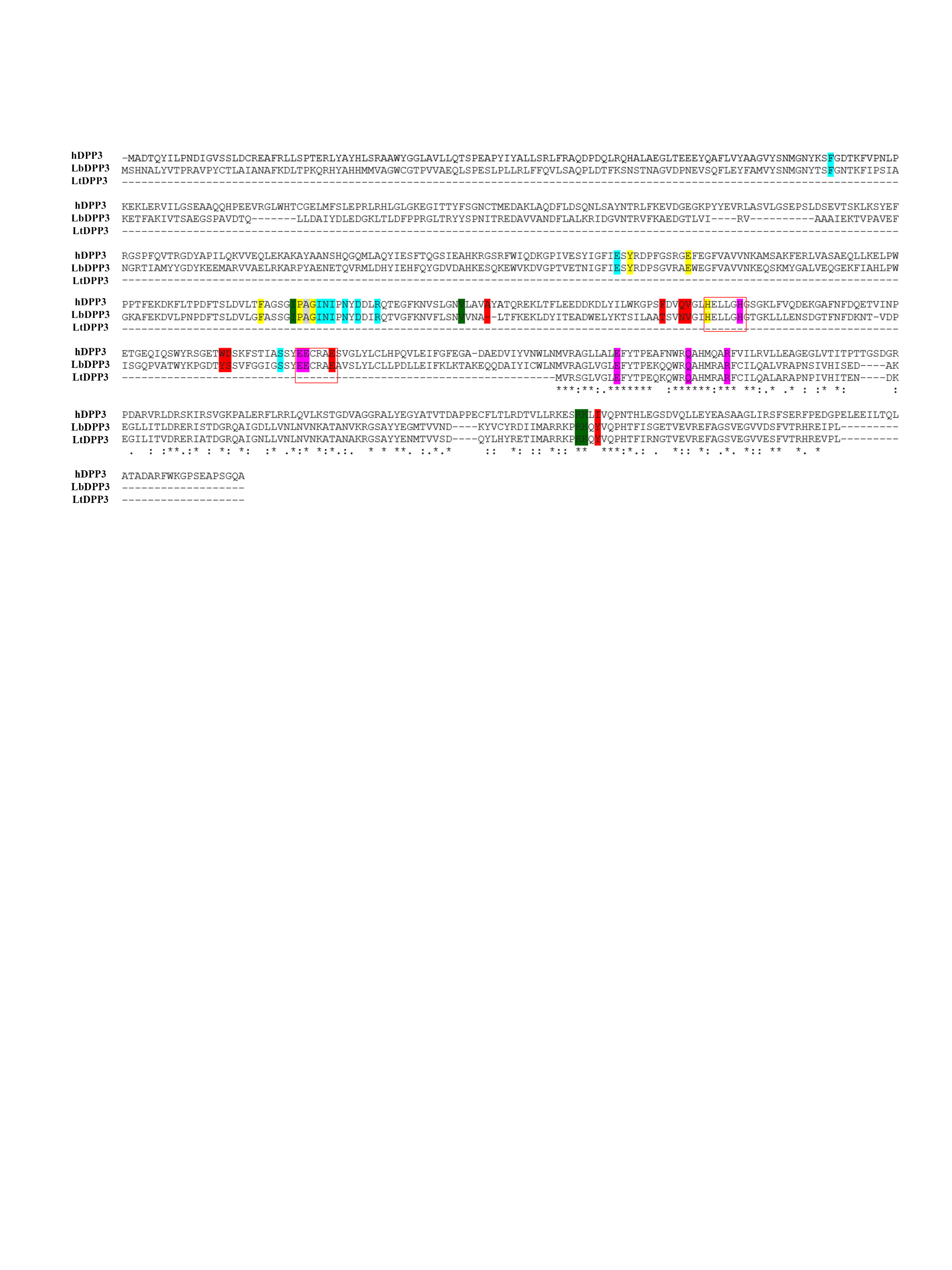

Supplement: S7 Fig — The boxes represent the conserved HELLGH and EECRAE motifs. Colors are used to represent the important amino acids for substrate binding as described previously (23). (TIF) [file pone.0190618.s007.TIF]
