# Supplementary material for: Dipeptidyl peptidase 3, a novel protease from Leishmania braziliensis
Source: PLoS One. 2018 Jan 5;13(1):e0190618. doi: 10.1371/journal.pone.0190618 (PMC5755878; doi:10.1371/journal.pone.0190618)
Supplement: S1 Table — (PDF) [file pone.0190618.s008.pdf]

Organism	Protein name	ID	Length (aa)	Cover %	Identity %
Leishmania aethiopica	metallo-peptidase, Clan M-, Family M49	LAEL147_000069200.1	679	100	88
Leishmania arabica	metallo-peptidase, Clan M-, Family M49 | protein	LARLEM1108_050014400.1	679	100	89
Leishmania donovani	dipeptidyl-peptidase III, putative	LdBPK_050960.1.1	679	100	89
Leishmania enriettii	metallo-peptidase, Clan M-, Family M49	LENLEM3045_050014900.1	679	100	89
Leishmania gerbilli	metallo-peptidase, Clan M-, Family M49	LGELEM452_050014500.1	679	100	89
Leishmania infantum	dipeptidyl-peptidase III, putative	LinJ.05.0960:mRNA	679	100	89
Leishmania major	metallo-peptidase, Clan M-, Family M49	LmjF.05.0960:mRNA	679	100	88
Leishmania mexicana	dipeptidyl-peptidase III, putative	LmxM.05.0960.1	679	100	89
Leishmania panamensis	metallo-peptidase, Clan M-, Family M49	LPAL13_050014300.1	679	100	99
Leishmania sp.	metallo-peptidase, Clan M-, Family M49	LMARLEM2494_050014800.1	679	100	87
Leishmania tarentolae	Dipeptidyl-peptidase III, putative	LtaP05.1040	156	22	84
Leishmania tropica	metallo-peptidase, Clan M-, Family M49	LTRL590_050014100.1	679	100	89
Leishmania turanica	metallo-peptidase, Clan M-, Family M49	LTULEM423_050014200.1	679	100	89
Leptomonas pyrrhocoris	dipeptidyl-peptidase III, putative,metallo-peptidase, Clan M-, Family M49	LpyrH10_37_0220	679	100	80
Endotrypanum monterogeii	Metallo-peptidase, Clan M-, Family M49	EMOLV88_050014200.1	679	100	80

Blechomonas ayalai	Dipeptidyl-peptidase III, putative	Baya_024_0020-1	681	100	65
Crithidia fasciculata	Metallo-peptidase, Clan M-, Family M49	CFAC1_020017600.1	679	100	80
Leptomonas seymouri	dipeptidyl-peptidase III putative metallo-peptidase Clan M- Family M49	Lsey_0296_0060-1	681	100	81
